# Safety and immunogenicity of the quadrivalent human papillomavirus vaccine in patients with juvenile dermatomyositis: a real-world multicentre study

**DOI:** 10.1186/s12969-020-00479-w

**Published:** 2020-11-11

**Authors:** Ingrid Herta Rotstein Grein, Natalia Balera Ferreira Pinto, Noortje Groot, Camila Bertini Martins, Aline Lobo, Nadia Emi Aikawa, Cassia Barbosa, Maria Teresa Terreri, Aline Coelho Moreira da Fraga, Sheila Knupp Feitosa de Oliveira, Flavio Sztajnbok, Luciana B. Paim Marques, Aline Garcia Islabão, Simone Appenzeller, Blanca Bica, Juliana de Oliveira Sato, Claudia Saad Magalhães, Virgínia Ferriani, Hella Pasmans, Rutger Schepp, Fiona van der Klis, Sytze de Roock, Nico Wulffraat, Gecilmara Salviato Pileggi

**Affiliations:** 1 Department of Paediatric Immunology and Rheumatology, Wilhelmina Children’s Hospital, University Medical Centre Utrecht, Utrecht, The Netherlands; 2 Department of Paediatrics, Hospital de Clínicas da Universidade Federal do Paraná, General Carneiro Street 68, 181, Alto da Gloria, Curitiba, PR 80060-900 Brazil; 3 Department of Paediatrics, Faculdade de Medicina de Ribeirão Preto, Universidade de São Paulo, São Paulo, Brazil; 4 Department of Preventive Medicine, Universidade Federal de São Paulo, São Paulo, Brazil; 5 Department of Paediatric Rheumatology, Instituto da Criança do Hospital das Clínicas da Faculdade de Medicina, Universidade de São Paulo, São Paulo, Brazil; 6 Department of Paediatric Rheumatology, Universidade Federal de São Paulo, São Paulo, Brazil; 7 Department of Paediatric Rheumatology, Hospital Estadual Infantil Nossa Senhora da Glória, Vitória, Brazil; 8 Department of Paediatric Rheumatology, Instituto de Puericultura e Pediatria Martagão Gesteira (IPPMG), Universidade Federal do Rio de Janeiro, Rio de Janeiro, Brazil; 9 Department of Paediatric Rheumatology, Universidade Estadual do Rio de Janeiro, Rio de Janeiro, Brazil; 10 Department of Paediatric Rheumatology, Hospital Infantil Albert Sabin, Fortaleza, Brazil; 11 Department of Paediatric Immunology and Rheumatology, University of Florida, College of Medicine, Florida, USA; 12 Department of Paediatric Rheumatology, Hospital da Criança de Brasília José Alencar, Brasília, Brazil; 13 Department of Paediatric Rheumatology, Universidade Estadual de Campinas, São Paulo, Brazil; 14 Department of Paediatric Rheumatology, Universidade Federal do Rio de Janeiro, Rio de Janeiro, Brazil; 15 Department of Paediatric Rheumatology, Universidade Estadual Paulista, Botucatu, São Paulo, Brazil; 16 Department of Paediatric Rheumatology, Faculdade de Medicina de Ribeirão Preto, Universidade de São Paulo, São Paulo, Brazil; 17 Centre for Infectious Disease Control, National Institute of Public Health and the Environment (RIVM), Bilthoven, The Netherlands; 18 Faculdade de Ciências da Saude Dr Paulo Prata (FACISB) e Instituto de Ensino e Pesquisa (IEP), Hospital de Câncer de Barretos, São Paulo, Brazil

**Keywords:** Juvenile dermatomyositis, Quadrivalent HPV vaccine, Safety, Immunogenicity

## Abstract

**Background:**

Concerns about the safety and efficacy of vaccines in patients with autoimmune diseases (AID) have led to contradictions and low vaccination coverage in this population, who are at a higher risk of infections, including by human papillomavirus (HPV). Although HPV vaccines have been recommended for immunocompromised patients, there is still a lack of data to support its use for AID patients, such as juvenile dermatomyositis (JDM) patients. The aim of this study was to assess the safety and immunogenicity of the quadrivalent HPV (qHPV) vaccine in a cohort of JDM patients.

**Methods:**

JDM patients aged from 9 to 20 years and healthy controls (HC) were enrolled to receive a 3-dose schedule of qHPV vaccine from March/2014 to March/2016. Study visits were performed before the first dose, 1 month after the second and third doses, and 6 months after the third dose. Participants completed a diary of possible adverse events for 14 days following each dose of vaccination (AEFV). Disease activity and current therapy were analyzed at each visit for JDM patients. In addition, serum samples from all participants were collected to test antibody concentrations against HPV16 and 18 at each visit. Participant recruitment was conducted in ten Brazilian centres. From 47 eligible JDM patients and 41 HC, 42 and 35, respectively, completed the 3-dose schedule of the vaccine, given that five JDM patients and two HC had received doses prior to their inclusion in the study.

**Results:**

The AEFVs presented by the participants were mild and in general did not differ between JDM and HC groups. No severe AEFVs were related to the vaccination. Disease activity was stable, or even improved during the follow-up. One month after the third dose of the vaccine the JDM group presented seropositivity of 100% for HPV16 and 97% for HPV18, similarly to the HC group, who presented 100% for both serotypes (*p* = 1.000). Six months after the third dose the seropositivity for the patient group was 94% for both HPV types.

**Conclusions:**

The HPV vaccination in this cohort of JDM patients was safe and immunogenic. Since the seropositivity against HPV16 and 18 was very high after the 3-dose schedule, this regimen should be recommended for JDM patients.

**Trial registration:**

Brazilian Clinical Trials Registry, number: RBR-9ypbtf. Registered 20 March 2018 – Retrospectively registered.

## Background

Numerous studies have shown that patients with autoimmune diseases (AID) are at a higher risk of infections and related complications due to the immunosuppression caused by the disease itself, associated with the use of medications that interfere with the immune system response [1–7]. The human papillomavirus (HPV) infection has been studied in this population, especially in patients with adult and juvenile systemic lupus erythematosus (SLE), the prototype of AID. It is known that SLE patients have a heightened risk of HPV infections and its complications such as genital warts and oncogenic lesions [8–13]. Patients with intestinal bowel disease (IDB) and juvenile idiopathic arthritis (JIA) were also described to be at a higher risk of HPV infections and cervical neoplasia [8]. Although no data are available regarding HPV infection in adult and juvenile dermatomyositis (JDM) patients, it is known that they are also more prone to complications and mortality from serious and opportunistic cutaneous and systemic infections [14].

Despite vaccination is the most effective resource to prevent infections, there are concerns regarding the safety and efficacy of vaccines related to AID and their immunosuppressive treatments, such as a potential risk of causing an exacerbation of the underlying disease, a possibility of inducing an infection (in case of a live-attenuated vaccine), or inefficacy due to the impaired immune response [1–7, 9]. The uncertainties surrounding this issue may directly impact on the vaccination cover in this group of patients [2, 15–18]. Interestingly, the main reason for the low vaccination coverage in AID patients is the lack of recommendation by physicians [15, 16].

HPV vaccines have been shown to be safe, well tolerated, and highly efficacious against vaccine-type HPV infection and its complications [19–22]. However, concerns regarding their safety have been raised after the publication of case reports about possible correlations between this vaccine and the onset of autoimmune and neurological conditions in healthy populations [23–25]. Epidemiological studies have been performed to further investigate this issue and this correlation was not confirmed [26, 27]. Indeed, the vaccine is considered safe by the World Health Organization (WHO) [28]. Moreover, in patients with pre-existing AID, some studies have shown that HPV vaccines were not associated with an increased incidence of new-onset AID [29, 30].

Quadrivalent HPV (qHPV) vaccine, Gardasil, is a non-live vaccine that immunizes against the main HPV subtypes (6, 11, 16 and 18). HPV 6 and 11 subtypes are responsible for the development of 90% of condylomata acuminate, whereas HPV 16 and 18 subtypes account for approximately 70% of cases of cervical cancer, 90% of anal cancer, 60% of vaginal cancer, and 50% of vulvar cancer worldwide. Gardasil has been widely used by healthy women from 9 to 26 years of age since its launch in 2006 in the United States of America and in Europe [19–22]. In Brazil, it was implemented in National Immunization Programs (NIP) in 2014, initially to girls from 11 to 13 years of age in a 3-dose schedule (0, 6 months, and 5 years). During the following years the vaccination schedule was changed and the immunization coverage was progressively extended. Currently in Brazil, the vaccination schedule comprises two doses (0 and 6 months) for healthy girls from nine to 14 years of age and for healthy boys from 11 to 14 years of age. The vaccine is recommended to immunocompromised patients from nine to 26 years of age in a 3-dose schedule (0, 2, and 6 months) [31, 32].

Although there is a recommendation for the administration of HPV vaccines for AID patients, there is still a lack of studies in patients with AID to support its safety and immunogenicity, especially among AID different from SLE and among the pediatric population [1–9]. The aim of this study was to evaluate the safety and immunogenicity of the qHPV vaccine in a multicentre Brazilian prospective study involving JDM female patients.

## Methods

This was a multicentre prospective controlled observational cohort study adapted from the Dutch protocol used to study the safety and immunogenicity of the bivalent HPV vaccine in children with JIA (68 patients), childhood SLE (six patients), and JDM (six patients) with a real-world approach [33, 34].

In the present study, a 3-dose schedule (0, 1 or 2, and 6 months) of the qHPV vaccine (against HPV6, HPV11, HPV16, HPV18) was used in patients who met the Bohan and Peter’s criteria for JDM [35], from 9 to 20 years of age, and healthy controls (HC). The doses of the qHPV vaccine used in the study were received by donation from the local Special Immunobiological Reference Centers of NIP.

Participants who were eligible and willing to receive the qHPV vaccine were enrolled in the study from March 2014 until March 2016. Moreover, JDM patients who had already received one or two doses of the qHPV vaccine before inclusion in the study were also allowed to participate, as a care standard to reach three doses, which is indicated for immunosuppressed patients [31, 32], since this is a real-life study.

Patients were recruited in ten pediatric rheumatology units from tertiary centres of different Brazilian’s regions. All JDM patients that attended their regular outpatient visits during the period of the study were invited to participate, regardless of disease activity or medication used, to constitute a real-life setting. Only patients with a new diagnosis were not invited to participate. Age and sex-matched HC were recruited from patient peer groups in two Brazilian study sites, composed of healthy girls who were friends or relatives of the recruited JDM patients and had a similar socioeconomic level to them. Study visits were planned before the first dose and 1 month after the second and third doses. JDM patients performed one more visit 6 months after the third dose. The protocol under code U1111–1211-2150, was approved by all the local ethics committees and informed consent was obtained from each participant and their guardians.

## Main outcome measures

To evaluate the safety of the qHPV vaccine, participants were asked to complete a diary for 14 days after each dose, about the occurrence of possible local and/or systemic adverse events following vaccination (AEFV). The local AEFV addressed included redness, bruising, edema, induration, and pain. Systemic AEFV included fever, skin abnormalities, itchiness, headache, nausea, vomiting, fatigue, fainting, and muscular and articular pain. In case of a severe AEFV, characterized by the WHO as an event that is life-threatening, requires in-patient hospitalization or prolongation of existing hospitalization, results in persistent or significant disability/incapacity, requires intervention to prevent permanent impairment or damage [36], patients were advised to go to the tertiary centre immediately to be evaluated by the investigating physician.

Another outcome considered for the safety evaluation included the assessment of disease activity in JDM patients at each study visit, using muscular and cutaneous parameters. For muscular evaluation the Childhood Myositis Activity Score (CMAS) and the Manual Muscle Testing (MMT) were used. The CMAS ranges from 0 (high disease activity) to 52 (no disease activity) [37], and the MMT ranges from 0 (high disease activity) to 80 (no disease activity) [38]. Disease was considered inactive when CMAS ≥ 48 and MMT ≥ 78 associated with satisfactory physician global assessment of overall disease activity (PhyGloVAS) [39, 40]. Based on these parameters, JDM patients were initially divided into three groups: “A” encompassed patients who had inactive disease at study inclusion, without using any medication; “B” encompassed patients who had inactive disease at inclusion, however were still using an immunosuppressive medication to control it; and “C” encompassed patients who had active disease at inclusion and were using immunosuppressive medications. To verify whether disease activity had changed after vaccination, the CMAS and/or MMT values were compared between visits, considering the disease as stable if the scores changed less than 20%; worsening if the scores decreased at least 20%; and improvement when both scores increased at least 20%. The variation of 20% was chosen based on the validated data-driven provisional criteria of the Paediatric Rheumatology International Trials Organisation (PRINTO) for the evaluation of response to therapy in JDM [41]. The most common cutaneous manifestations of JDM (cutaneous rash, heliotrope of the upper eyelids, and Gottron’s papules) were evaluated on each visit according to their intensity and compared as follows: improvement, if the manifestation has subsided; stable, if it remained unchanged; worsening, if it had aggravated. In order to reduce the evaluation bias, the analysis of the patient’s skin was performed by the same experienced pediatric rheumatologist on all visits. Comparison of the medications in use at each visit, which can indirectly quantity disease activity intensity, was used as an additional parameter, as follows: stable disease, if the medication remained the same between the visits; improvement, if it had been withdrawn; worsening, if a new treatment had been added or if previous treatment doses had been increased. In order to evaluate the changes in the measured values, considering CMAS and MMT scores, cutaneous manifestations, and use of medications, according to the established criteria already specified, comparisons were made between visits.

As good practice in clinical trials, all participants received the investigators’ contact details and were guided to contact the hospital if any symptom occurred during the study period, or in case of any doubt regarding the study protocol. Each participant centre had the autonomy to decide whether their patients would continue to receive the qHPV vaccination in case of disease worsening or severe AEFV.

For immunogenicity evaluation, blood drawing was performed at each study visit. Serum was collected and frozen under − 70 °C in Brazil and subsequently shipped to the Netherlands for serologic antibody concentration testing using a virus-like particle based multiplex Luminex assay [33]. Seropositivity for HPV16 and HPV18 was defined as an antibody concentration higher than 9 Luminex Units/ml and 13 Luminex Units/ml, respectively.

The qualitative variables are presented as absolute frequencies and percentages, whereas the quantitative variables are presented as medians and interquartile ranges (IQR). The Fisher’s exact test was used for comparisons between two independent qualitative variables using 2 × 2 contingency tables. The chi-squared test with Monte Carlo simulation was used for analysis of all other qualitative variables. The comparisons of the quantitative variables between two or three groups were performed with the Wilcoxon-Mann-Whitney test and Kruskal-Wallis test, respectively. CMAS at month X was compared with month Y using the non-parametric Wilcoxon matched pair test. The binary variables (rash, Gottron’s papules, and heliotrope) were calculated at baseline and after the third dose of the vaccine using the McNemar test, considering two possible outcomes: disease stability or worsening. Statistical significance was defined as a *p* value of < 0.05. The statistical analysis were performed using software R, version 3.6.1.

## Results

**Fig. 1:**
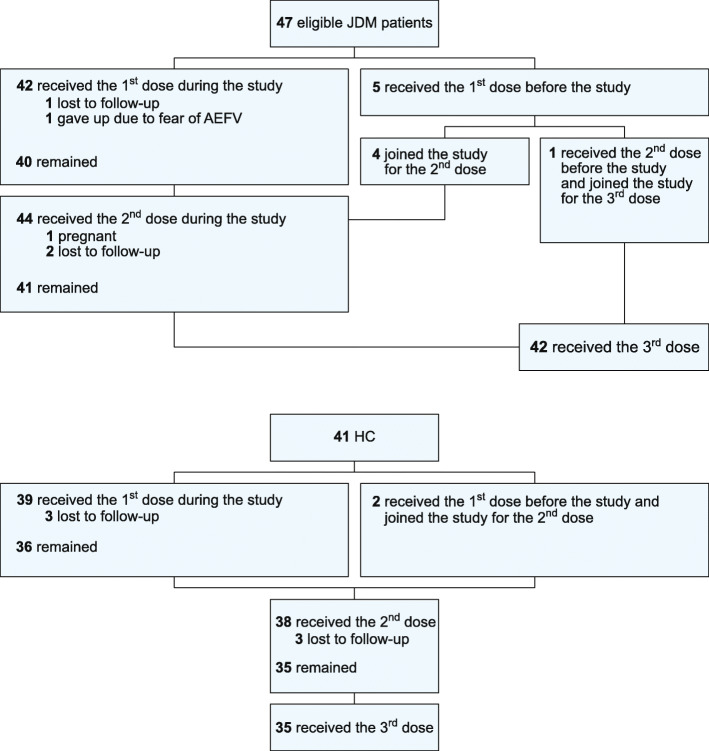
Flow diagram describing qHPV vaccine administration in JDM patients and HC before and during the study. JDM: juvenile dermatomyositis; AEFV: adverse events following vaccine; HC: healthy controls

Forty-seven JDM patients and 41 HC were initially eligible for the study. Four JDM patients and two participants from the HC group had previously received one dose of the vaccine. One JDM patient had received two doses. During the study period, five patients did not receive all three doses of the vaccine: three due to lack of follow-up, one patient due to fear of AEFV, and the last patient became pregnant after receiving two doses. Forty-two JDM patients and 35 HC completed the 3-dose schedule. The flow diagram describing the administration of the qHPV vaccine doses prior to and during the study is presented in Fig. 1.

Characteristics of the participants at study inclusion are summarized in Table 1. Median age at diagnosis of JDM was 7 years. Median age at first dose of qHPV vaccine was 16 for JDM patients and 14 for HC. Twelve/47 (25.5%) patients had inactive disease at study inclusion and were not using any immunosuppressive medication (JDM group A). Thirty-five/47 (74.5%) JDM patients used at least one medication for disease control at study inclusion, divided between 17/47 (36.2%) patients with inactive disease (JDM group B) and 18/47 (38.3%) with active disease despite the use of immunosuppressive medications (JDM group C). Nine/47 (19.1%) JDM patients and 11/47 (26.8%) HC reported having initiated sexual activity before their inclusion in the study. Baseline blood samples were collected from 37 JDM patients and 39 HC. Baseline samples were not taken from the participants who had been vaccinated before the study. At baseline, two/39 (5%) HC were seropositive for HPV16 and one/39 (3%) for HPV18. Seropositivity at baseline for JDM patients was 10/37 (27%) for HPV16, and 9/37 (24%) for HPV18. Of the 13 patients who were seropositive for HPV 16 and/or HPV18 at baseline, only two reported being sexually active at the time of the study.

**Table 1:** Characteristics of JDM patients and HC at study inclusion

	**JDM (*n*** **= 47)**	**HC (*n*** **= 41)**	***p*-value**^**c**^
First qHPV vaccine dose between 9 to 13 years, n (%)	20 (42.6)	9 (22.0)	0.045
First qHPV vaccine dose between 14 to 20 years, n (%)	27 (57.4)	32 (78.0)	0.045
First qHPV vaccine dose, median age in years (IQR)	16 (13.0–19.0)	14 (6.5–21.5)	0.001
Diagnosis, median age in years (IQR)	7 (0–14.5)	NA	NA
1 dose previous to the study, n (%)	4 (8.5)	2 (4.9)	1.000
2 doses previous to the study, n (%)	1 (2.1)	0 (0.0)	1.000
Sexual activity initiation, n (%)	9 (19.1)	11 (26.8)	0.450
Immunossupressive medications use, n (%)	35 (74.5)	NA	NA
JDM group A^a^, n (%) [median CMAS]	12 (25.5) [52]	NA	NA
JDM group B^a^, n (%) [median CMAS]	17 (36.2) [52]	NA	NA
JDM group C^a^, n (%) [median CMAS]	18 (38.3) [37]	NA	NA
	**JDM (*n*** **= 37)**	**HC (*n*** **= 39)**	***p*-value**^**c**^
Seropositivity for HPV 16, n (%)	10 (27.0)^b^	2 (5.1)	0.011
Seropositivity for HPV 18, n (%)	9 (24.3)^b^	1 (2.6)	0.006

*JDM* juvenile dermatomyositis, *HC* healthy controls, *HPV* human papillomavirus, *qHPV* quadrivalent HPV vaccine, *NA* not applicable, *CMAS* childhood myositis activity score, *IQR* interquartile range

^a^JDM groups: A = inactive disease without medication; B = inactive disease with medication; C = active disease with medication. Considering active disease: CMAS < 48 or MMT < 78 or unsatisfactory physician global assessment of overall disease activity (PhyGloVAS)

^b^Six patients were seropositive for both HPV16 and 18, four were seropositive only for HPV16, and three only for HPV18

^c^Statistical significance was defined as a *p*-value < 0.05

Concerning AEFV evaluation, a total of 121 diaries (40 after the first dose, 41 after the second dose, and 40 after the third dose) from 47 JDM patients were analyzed, as well as 111 diaries (38 after the first and second doses and 35 after the third dose) from 41 HC individuals. Pain was the most common local adverse event reported for both JDM patients (55%) and HC (60.5%) after the first dose (*p* = 0.653). After the third dose, despite the decrease in frequency, local pain remained the most common local symptom reported by 40% of the JDM group and 54.3% of the HC (*p* = 0.252). The frequency of all other local symptoms was similar between both groups, except for bruise and edema, which were more frequent among the HC group after receiving the third vaccine dose (*p* = 0.019 and *p* = 0.010, respectively). Headache was the most frequently reported systemic adverse event after all three HPV doses among patients and HC (*p* 0.534), followed by fatigue (*p* = 0.263). JDM patients presented significantly more nausea after the first dose (*p* = 0.014) and more itchiness after the third dose (*p* = 0.013) than HC. No severe adverse events were related to the vaccination. The majority of AEFV lasted only 1 or 2 days and almost none more than 7 days. The occurrence of AEFV and related statistical analysis are described in Table 2.

**Table 2:** Ocurrence of AEFV in JDM patients and HC after each qHPV vaccine dose

	After the first dose			After the second dose			After the third dose			Total		
	JDM	HC	*p*-value^b^	JDM	HC	*p*-value^b^	JDM	HC	*p*-value^b^	JDM	HC	*p*-value^b^
**Vaccinated patients during the study, n**	**42**	**39**	**NA**	**44**	**38**	**NA**	**42**	**35**	**NA**	**128**	**112**	NA
**Completed diaries after vaccine dose, n (%)**	**40 (95.2)**	**38 (97.4)**	**NA**	**41 (93.2)**	**38 (100)**	**NA**	**40 (95.2)**	**35 (100)**	**NA**	**121 (94.5)**	**111 (99.1)**	NA
**Local AEFV, n (%)**												
Redness	5 (12.5)	4 (10.5)	1.000	2 (4.9)	3 (7.9)	0.667	0 (0.0)	2 (5.7)	0.214	7 (5.8)	9 (8.1)	0.601
Bruise	0 (0.0)	0 (0.0)	1.000	1 (2.4)	1 (2.6)	1.000	0 (0.0)	5 (14.3)	0.019	1 (0.8)	6 (5.4)	0.058
Edema	5 (12.5)	5 (13.2)	1.000	4 (9.8)	8 (21.1)	0.215	1 (2.5)	8 (22.9)	0.010	10 (8.3)	21 (18.9)	0.021
Induration	6 (15.0)	9 (23.7)	0.396	4 (9.8)	4 (10.5)	1.000	4 (10.0)	5 (14.3)	0.726	14 (11.6)	18 (16.2)	0.346
Pain	22 (55.0)	23 (60.5)	0.653	19 (46.3)	23 (60.5)	0.261	16 (40.0)	19 (54.3)	0.252	57 (47.1)	65 (58.6)	0.115
**Systemic AEFV, n (%)**												
Fever	1 (2.5)	0 (0.0)	1.000	1 (2.4)	0 (0.0)	1.000	0 (0.0)	1 (2.9)	0.467	2 (1.7)	1 (0.9)	1.000
New cutaneous abnormalities^a^	2 (5.0)	0 (0.0)	0.494	1 (2.4)	0 (0.0)	1.000	1 (2.5)	0 (0.0)	1.000	4 (3.3)	0 (0.0)	0.052
Itchiness	1 (2.5)	1 (2.6)	1.000	3 (7.3)	1 (2.6)	0.616	7 (17.5)	0 (0.0)	0.013	11 (9.1)	2 (1.8)	0.020
Headache	9 (22.5)	10 (26.3)	0.794	10 (24.4)	10 (26.3)	1.000	6 (15.0)	7 (20.0)	0.761	25 (20.7)	27 (24.3)	0.534
Nausea	9 (22.5)	1 (2.6)	0.014	1 (2.4)	2 (5.3)	0.606	2 (5.0)	4 (11.4)	0.409	12 (9.9)	7 (6.3)	0.346
Vomiting	2 (5.0)	0 (0.0)	0.494	0 (0.0)	0 (0.0)	1.000	0 (0.0)	0 (0.0)	1.000	2 (1.7)	0 (0.0)	0.498
Fatigue	6 (15.0)	7 (18.4)	0.767	4 (9.8)	7 (18.4)	0.338	4 (10.0)	5 (14.3)	0.726	14 (11.6)	19 (17.1)	0.263
Fainting	0 (0.0)	0 (0.0)	1.000	0 (0.0)	0 (0.0)	1.000	0 (0.0)	0 (0.0)	1.000	0 (0.0)	0 (0.0)	1.000
Initial or worsened muscular pain	2 (5.0)	1 (2.6)	1.000	2 (4.9)	1 (2.6)	1.000	0 (0.0)	0 (0.0)	1.000	4 (3.3)	2 (1.8)	0.685
Initial or worsened articular pain	1 (2.5)	0 (0.0)	1.000	1 (2.4)	0 (0.0)	1.000	0 (0.0)	0 (0.0)	1.000	2 (1.7)	0 (0.0)	0.499
**Severe AEFV, n (%)**												
WHO definition	0 (0.0)	0 (0.0)	1.000	0 (0.0)	0 (0.0)	1.000	0 (0.0)	0 (0.0)	1.000	0 (0.0)	0 (0.0)	1.000

*JDM* juvenile dermatomyositis, *HC* healthy controls, *AEFV* adverse events following vaccination, *qHPV* quadrivalent human papillomavirus vaccine, *NA* Not applicable, *WHO* World Health Organization

^a^Patients described new rash on face or on body, that subsided in a maximum of 4 days

^b^Statistical significance was defined as a *p*-value < 0.05

In total, JDM patients had 42 baseline visits, 42 visits after the second dose, 40 visits after the third dose, and 26 visits 6 months after the third dose. Table 3 shows the muscular and cutaneous disease activity evaluation and the medication in use by JDM patients at each study visit. All JDM patients were evaluated by the CMAS score, whereas the MMT score was performed only by a few investigators. Consequently, the CMAS was the main score for muscular evaluation in this study. The median of disease activity measured through the CMAS score for the JDM population was 50 at the first visit, 51.5 at the second visit, and 50 at the third and fourth visits. The analysis of disease activity among the JDM groups A, B, and C revealed a median CMAS of 52 for groups A and B at the four visits, whereas group C presented a median CMAS of 37, 42, 46, and 43 at the four visits, respectively. Five patients from group C (27.8% patients from this group) demonstrated an improvement greater than 20% in their CMAS (CMAS scores increased 15 to 20 points in these five patients right after the third dose compared with baseline). The analysis of disease activity performed 6 months after the end of the 3-dose schedule showed that two of the five patients, who had significantly improved, presented worsening scores in comparison with the scores presented immediately after receiving the third dose. One of these had a CMAS score of 48 after the third dose. Due to her excellent improvement, one of her medications was suspended (cyclosporin). Unfortunately, at the last visit she had returned to her baseline score (CMAS 28). The other patient also scored CMAS 48 after receiving the third dose, however at the final visit ended with only two extra points compared to baseline (final CMAS 31). One patient from group B presented worsening in scores of greater than 20% in the fourth visit and maintained a final score lower than her baseline score (CMAS at first, second, and third visits: 48; CMAS at fourth visit: 32). This patient had her only medication (cyclosporin) suspended during the study. The changes in CMAS are presented in Table 4. There was no significant worsening regarding cutaneous manifestation between the baseline and after the third vaccine dose (*p* = 0.074 for rash; *p* = 0.814 for Gottron’s papules; *p* = 0.479 for heliotrope).

**Table 3:** Muscular and cutaneous activity evaluation and medications in use by JDM patients at each study visit

Study visits^a^	V1^b^	V2	V3	V4	V1^b^	V2	V3	V4	V1^b^	V2	V3	V4
JDM groups	A + B (*n* = 27)	A + B (*n* = 27)	A + B (*n* = 25)	A + B (*n* = 20)	C (*n* = 15)	C (*n* = 15)	C (*n* = 15)	C (*n* = 6)	Total (*n* = 42)	Total (*n* = 42)	Total (*n* = 40)	Total (*n* = 26)
**Muscular activity**												
CMAS, median	52	52	52	52	37	42	46	43	50	51,5	50	50
**Cutaneous activity**												
Rash, n (%)	0 (0.0)	0 (0.0)	0 (0.0)	0 (0.0)	9 (60)	7 (46.7)	4 (26.7)	1 (16.7)	9 (21.4)	7 (16.7)	4 (10)	1 (3.8)
Gottron’s papules, n (%)	0 (0.0)	0 (0.0)	1 (4.0)	0 (0.0)	12 (80)	9 (60)	9 (60)	3 (50)	12 (28.6)	9 (21.4)	10 (25)	3 (11.5)
Heliotropo, n (%)	0 (0.0)	0 (0.0)	0 (0.0)	0 (0.0)	7 (46.7)	4 (26.7)	5 (33.3)	1 (16.7)	7 (16.7)	4 (9.5)	5 (12.5)	1 (3.8)
**Medications in use**												
Costicosteroids, n (%)	5 (18.8)	5 (18.5)	5 (20.0)	5 (25.0)	15 (100)	15 (100)	15 (100)	6 (100)	20 (47.6)	20 (47.6)	19	11 (42.3)
Oral Prednisone, n (%) [median dose]	5 (18.5) [15]	5 (18.5) [5]	5 (20.0) [10]	5 (25.0) [5]	12 (80) [20]	13 (86.7) [10]	12 (80) [10]	5 (83.3) [10]	17 (40,5) [15]	18 (42.9) [10]	16 (40) [10]	10 (38.5) [6.25]
IV Methylprednisolone, n (%)	0 (0.0)	0 (0.0)	0 (0.0)	0 (0.0)	5 (33.3)	3 (20)	3 (20)	1 (16.7)	5 (11.9)	3 (7.1)	3 (7,5)	1 (3.8)
Hydroxychloroquine, n (%)	9 (33.3)	8 (29.6)	8 (32.0)	4 (20.0)	8 (53.3)	10 (66.7)	10 (66.7)	4 (66.7)	17 (40.5)	18 (42.9)	18 (45)	8 (30.8)
Methotrexate, n (%)	6 (22.2)	5 (18.8)	6 (24.0)	4 (20.0)	9 (60)	10 (66.7)	9 (60)	3 (50)	15 (35.7)	15 (35.7)	15 (37.5)	7 (26.9)
Azathioprine, n (%)	5 (18.8)	4 (14.8)	5 (20.0)	3 (15.0)	3 (20)	3 (20)	3 (20)	2 (33.3)	8 (19)	7 (16.7)	8 (20)	5 (19.2)
Mycophenolate Mofetil, n (%)	0 (0.0)	0 (0.0)	0 (0.0)	0 (0.0)	1 (6.7)	1 (6.7)	2 (13.3)	1 (16.7)	1 (2.4)	1 (2.4)	2 (5)	1 (3.8)
Cyclosporine, n (%)	3 (11.1)	3 (11.1)	3 (12.0)	3 (15.0)	4 (26.7)	4 (26.7)	3 (20)	0 (0.0)	7 (16.7)	7 (16.7)	6 (15)	3 (11.5)
Cyclophosphamide, n (%)	0 (0.0)	0 (0.0)	0 (0.0)	0 (0.0)	1 (6.7)	1 (6.7)	0 (0.0)	0 (0.0)	1 (2.4)	1 (2.4)	0 (0.0)	0 (0.0)
Human Immunoglobulin, n (%)	0 (0.0)	0 (0.0)	0 (0.0)	0 (0.0)	2 (13.3)	2 (13.3)	0 (0.0)	0 (0.0)	2 (4.8)	2 (4.8)	0 (0.0)	0 (0.0)
No medication, n (%)	11 (40.7)	13 (48.1)	11 (44.0)	8 (40.0)	0 (0.0)	0 (0.0)	0 (0.0)	0 (0.0)	11 (26,2)	13 (31)	11 (27.5)	8 (30.8)

*JDM* juvenile dermatomyositis, *qHPV* quadrivalent human papillomavirus vaccine, *CMAS* childhood myositis activity score, *IV* intravenous

^a^V1: baseline visit; V2: visit after the second dose; V3: visit after the third dose; V4: visit 6 months after the third dose

^b^Five patients who had received doses of the vaccine before the study inclusion were excluded from the analysis of the baseline visit

**Table 4:** Descriptive comparison of disease activity and medications in use at baseline and after the third qHPV vaccine dose

CMAS (*n* = 40)^a^		Cutaneous manifestations (*n* = 40)^a^				Medications in use (*n* = 40)^a^	
			Rash	Gottron’s papules	Heliotrope		
Definition	n (%)	Definition	n (%)	n (%)	n (%)	Definition	n (%)
Stable with CMAS ≥48	24 (60.0)	Stable without cutaneous lesion	31 (77.5)	27 (67.5)	33 (82.5)	Stable without medication	11 (27.5)
Stable with CMAS <48	11 (27.5)	Stable with cutaneous lesion	3 (7.5)	9 (22.5)	2 (5.0)	Stable with medication	9 (22.5)
CMAS Improvement	5 (12.5)	Improvement of cutaneous lesion	5 (12.5)	3 (7.5)	3 (7.5)	Decreased medication	14 (35.0)
CMAS Worsening	0 (0.0)	Worsening or new-onset of cutaneous lesion	1 (2.5)	1 (2.5)	2 (5.0)	Increased medication	6 (15.0)

*CMAS* Childhood Myositis Activity Score, *qHPV* quadrivalent human papillomavirus vaccine

^a^Data from 40 patients were available for comparison between the baseline visit and the visit after the third vaccine dose

Twelve/42 (28.6%) patients presented at least one typical cutaneous manifestation at baseline. Nine/42 (21.4%) had a cutaneous rash, 12/42 (28.6%) had Gottron’s papules, and seven/42 (16.7%) had heliotrope of the upper eyelids. Regarding the patients that had already presented a cutaneous abnormality at baseline, improvement in the lesion occurred in five/nine (55.5%) patients with cutaneous rash, three/12 (25%) with Gottron’s papules, and three/seven (37.5%) with heliotrope, after receiving three doses of the vaccine. Worsening occurred in one/nine (11.1%) patients with a rash and two/seven (28.6%) patients with heliotrope. Only one patient from group B had new-onset JDM cutaneous manifestations during the study. The changes in cutaneous manifestations are demonstrated in Table 4. There were no differences regarding cutaneous activity between the baseline and after receiving the third vaccine dose (*p* > 0.050 for all evaluated cutaneous manifestations).

Corticosteroids were the most commonly prescribed medication, used by 20/42 (47.6) of the JDM patients at baseline, with a median dose of 15 mg/day. Of the 20 patients who used corticosteroids, 15 were from group C (100% of the group) and five from group B (31.3% of the group). Hydroxychloroquine was the second most commonly prescribed medication, used by 17/42 (40.5%) of the patients at baseline, followed by methotrexate (35.7%), azathioprine (19%), and cyclosporine (16.7%). Two patients were using human immunoglobulin (IGIV) (4.8%). One patient was using mycophenolate mofetil, and one patient was using cyclophosphamide at baseline (2.4% each). Regarding the use of cyclophosphamide, two patients had recently used it before inclusion in the study (received the last dose 6 and 8 months before inclusion). These two patients belonged to group C.

Throughout the study, 27/40 patients (67.5%) maintained the same (stable) treatment. No patients from group A used any medication during the study period. Six/40 (15%) patients had their immunosuppressive treatment increased due to disease activity. One patient from group B started prednisone 15 mg/day after receiving three vaccine doses, due to cutaneous activity (her CMAS was 52 throughout the study). This patient’s prednisone dose was decreased to 5 mg/day at the last visit. Five patients from group C (27.8% of the group) had their immunosuppressive medication changed or had to initiate a new medication during the study, associated with an increase in the prednisone dose. However, these five patients already had active disease before receiving the initial qHPV dose (CMAS of these five JDM patients was between 37 and 46 at baseline). In three of these patients, the prednisone dose was decreased at the next study visit and one of them had IGIV suspended. Fourteen/40 (35%) patients had their immunosuppressive treatment decreased during the study. Three patients from group B and four patients from group C had one or two immunosuppressive medications withdrawn (three and four patients, respectively). Among the medications removed were prednisone (two patients), cyclophosphamide (one patient), and IGIV (one patient). In addition to these two patients who had prednisone withdrawn, the prednisone dose of nine more patients was decreased during the study. The main changes in the medications used by the patients are shown in Table 4. No statistical significance was found between the baseline and the visit after the third vaccine dose regarding drug therapy of the JDM patients (*p* = 1.000).

Thirty-six JDM patients received the first two doses of qHPV vaccine and had a blood sample collected after it. After the second dose, the seropositivity was 94 and 92% for HPV16 and HPV18, respectively. Two patients remained seronegative for both HPV types (one from group B and one from group C), and one patient remained seronegative for only HPV18 (group C). The patient from group B was using only methotrexate 25 mg/week. The patient from group C that remained seronegative for both HPV types was using prednisone 10 mg/day plus hydroxychloroquine. The patient that remained seronegative only for HPV18 was under a low dose of prednisone (7,5 mg/day) associated with hydroxychloroquine and azathioprine. The patient that was using cyclophosphamide during the study and the two patients who had recently used this medication demonstrated seropositivity for both HPV serotypes. The comparative analysis of the serological response after two doses of the vaccine did not demonstrate differences between the JDM groups A, B, and C (*p* = 1.000 for HPV16 and *p* = 0.770 for HPV18).

Seropositivity of the 31 JDM patients who completed the 3-dose schedule and had a blood sample collected after it was 100% for HPV16 and 97% for HPV18. Only one JDM patient remained seronegative for HPV18 immediately after receiving all three doses. This was the same patient who had already remained seronegative only for HPV18 after receiving two doses (group C). There were no differences between JDM groups A, B, and C regarding the comparative analysis of the serological response after three doses of the vaccine (*p* = 1.000 for both HPV types).

Blood samples were available for 17 JDM patients 6 months after the final dose. This analysis showed that 94% of the patients remained seropositive for HPV16 and HPV18. Only one patient, who had presented seropositive for HPV16 and 18 during the study, became seronegative for both HPV types during the following months. This patient had inactive disease and was only using cyclosporine during the study period (group B). Once more, JDM groups A, B, and C showed similar serological response to the vaccine (*p* = 1.000 for both HPV types).

The HC group responded to the vaccination after two and three doses, with 100% seropositivity for both HPV serotypes (samples available from 14 HC after two doses, and from 31 after three doses). No differences were observed in the comparative analysis of the serological response to vaccination between JDM patients and HC, either after two doses (*p* = 1.000 for HPV16 and *p* = 0.265 for HPV18), or after three doses (*p* = 1.000 for both HPV types). The serological analysis of JDM patients and HC is shown in Table 5. Samples were not collected from the HC group 6 months after the last dose.

**Table 5:** Seropositivity analysis of JDM patients and HC at each study visit

**Baseline visit**							
	**JDM A (*n*** **= 10)**	**JDM B (*n*** **= 13)**	**JDM C (*n*** **= 14)**	***p*-value**	**JDM Total (*n*** **= 37)**^**a**^	**HC (*n*** **= 39)**^**a**^	***p*-value**
**HPV 16, n (%)**	1 (10.0)	6 (46.0)	3 (21.0)	0.142	10 (27.0)	2 (5.0)	0.012
**HPV 18, n (%)**	1 (10.0)	4 (31.0)	4 (29.0)	0.571	9 (24.0)	1 (3.0)	0.006
**After the second qHPV vaccine dose**							
	**JDM A (*n*** **= 10)**	**JDM B (*n*** **= 11)**	**JDM C (*n*** **= 15)**	***p*-value**	**JDM Total (*n*** **= 36)**^**b**^	**HC (*n*** **= 14)**^**c**^	***p*-value**
**HPV 16, n (%)**	10 (100)	10 (91.0)	14 (93.0)	1.000	34 (94.0)	14 (100)	1.000
**HPV 18, n (%)**	10 (100)	10 (91.0)	13 (87.0)	0.770	33 (92.0)	14 (100)	0.265
**After the thrid qHPV vaccine dose**							
	**JDM A (*n*** **= 8)**	**JDM B (*n*** **= 11)**	**JDM C (*n*** **= 12)**	***p*-value**	**JDM Total (*n*** **= 31)**^**b**^	**HC (*n*** **= 31)**^**c**^	***p*-value**
**HPV 16, n (%)**	8 (100)	11 (100)	12 (100)	–	31 (100)	31 (100)	1.000
**HPV 18, n (%)**	8 (100)	11 (100)	11 (92.0)	1.000	30 (97.0)	31 (100)	1.000
**Six months after the third qHPV vaccine dose**							
	**JDM A (*n*** **= 5)**	**JDM B (*n*** **= 7)**	**JDM C (*n*** **= 5)**	***p*-value**	**JDM Total (*n*** **= 17)**^**b**^	**HC**	***p*-value**
**HPV 16, n (%)**	5 (100)	6 (86.0)	5 (100)	1.000	16 (94.0)	NA	NA
**HPV 18, n (%)**	5 (100)	6 (86.0)	5 (100)	1.000	16 (94.0)	NA	NA

*JDM* juvenile dermatomyositis, *HC* healthy controls, *HPV* human papillomavirus, *qHPV* quadrivalent HPV vaccine, *NA* not applicable

^a^Five patients and two HC who had received doses of the vaccine before the study inclusion were excluded from this analysis

^b^Forty-two JDM patients completed the 3-dose vaccination schedule, however blood samples were collected only from 36 patients after the second dose, 31 after the third dose, and 17 6 months after the third dose

^c^Thirty-five HC completed the 3-dose vaccination schedule; however, blood samples were collected only from 14 HC after the second dose, and 31 after the third dose

## Discussion

This is the largest prospective study addressing safety and immunogenicity of a qHPV in a pediatric population with JDM. This study is in a real life setting, where patients were included despite their disease activity and the use of glucocorticoids and/or immunosuppressive treatment. Even so, the qHPV vaccine was safe and immunogenic in this cohort of JDM patients.

The occurrence of AEFV in the 2 weeks following vaccination was similar between JDM patients and the HC group. All AEFV were mild, such as local pain, local edema and induration, headache, fatigue, or nausea, and presented spontaneous resolution shortly after the vaccination. In general, AEFV decreased throughout the study among both patient and HC groups. No severe adverse events were related to the vaccination.

The analysis of disease activity showed that the majority of patients remained stable throughout the study period, independent of their baseline activity. Interestingly, five patients from group C (active disease with immunosuppressive medication) even significantly improved during the study period (CMAS increased at least 20%). Unfortunately, two of these patients returned to their baseline CMAS 6 months later. Despite the fact that there were few patients with cutaneous manifestations in this study, the analysis showed that the majority of patients remained stable. In addition, a greater number of patients presented improvement in their lesions compared to those with worsening lesions during the study period. Another interesting aspect of this study is regarding the analysis of the immunosuppressive treatment of the JDM patients. Patients, especially those from group C, were using diverse immunosuppressive medications, such as oral and intravenous corticosteroids and cyclophosphamide. Throughout the study, the majority of patients maintained their initial treatment; six patients needed to have their immunosuppressive treatment increased, and 14 patients had their medication withdrawn or decreased.

The combined analysis of mild AEFV (similar to the HC group) and stable disease activity throughout the study showed that the 3-dose scheme of qHPV vaccine was safe in this JDM cohort. Our results are in accordance with other studies that showed no influence of HPV vaccines regarding AEFV and disease activity in patients with AID [8, 29, 30, 42–45].

The seropositivity of JDM patients for HPV16 and HPV18 after the 3-dose schedule was extremely high (almost 100%), even among patients who were using immunosuppressive therapy. The statistical analysis showed that there were no differences regarding the serological response between JDM groups A, B, and C, as well as between the total JDM cohort and the HC group. Interestingly, the only patient that remained seronegative for HPV18 was not the one with highest immunosuppressive treatment (she was using a low dose of prednisone, associated with hydroxychloroquine and azathioprine). Patients with a higher level of immunosuppression, such as intravenous corticosteroids and cyclophosphamide presented seropositivity for both serotypes. This result was surprisingly good, as it is generally accepted that patients using immunosuppressive drugs, especially at high doses, present a diminished response to vaccinations [3, 5, 6]. Our results are in accordance with other studies addressing HPV vaccinations in patients with AID [42–45]. In those studies, HPV-vaccination induced seroconversion in the large majority of patients. Although this was not a long-term follow-up study, it was shown that 6 months after the third qHPV dose the majority of patients from who blood was collected remained seropositive for HPV16 and18.

This study has some limitations. Although it is the largest reported cohort of JDM patients and qHPV vaccination to date, the number of participants in the study was still low. As JDM is a rare disease, it is extremely difficult to perform a study with a large number of patients. We demonstrated that the qHPV vaccine was safe in our cohort of patients, since we did not observe severe AEFV or disease worsening or flare-up. However, in order to analyze uncommon AEFV, it would be necessary to evaluate a much larger number of patients to entirely demonstrate the safety of the vaccine.

Regarding the serological analysis, a consideration has to be addressed about the high seropositivity rates for HPV16 and 18 at baseline, in contrast with the low seropositivity presented for the HC group. There is no clear explanation for this difference. Some hypotheses were raised, such as the possibility that some JDM patients did not report sexual activity or a previous HPV vaccination. Whatever the reason might be, these patients presented a sustained immunological response, and remained seropositive until the end of the study.

Another limitation of this study is that some patients missed their medical appointments and consequently their disease activity was not evaluated, and their blood samples were not collected on all visits. The last visit (6 months after the third vaccine dose) was the most affected. The low adherence to this visit may have impaired the final analysis of the study, as we do not know whether the missing patients presented maintained stable disease activity, and whether they remained seropositive or not. It would be very interesting to analyze the same cohort of patients some years in the future, and to study whether the seropositivity remained or not. It was recently shown that the majority of SLE patients retained the immunogenicity of the qHPV vaccine after 5 years, although patients who had more SLE flares and had received higher cumulative doses of certain immunosuppressive agents were at risk of sero-reversion of the anti-HPV antibodies [46]. Currently, there are no studies regarding this subject in JDM patients.

HPV vaccination was shown to have a safe profile and adequate immunogenicity in this cohort of JDM patients. The 3-dose regimen reached very high seropositivity for HPV16 and HPV18 (similar to the HC group) without inducing any flare-ups regarding disease activity or any severe AEFV. Therefore, this schedule should be recommended in this population with a high risk of developing oncogenic HPV. Long-term follow-up studies are still necessary to show the duration of protection against HPV infections in JDM patients and to assess the need for booster vaccinations. As there is no information in the literature regarding long-term protection in JDM patients, cervical smears should still be performed as secondary prevention of cervical abnormalities.

## Conclusion

Although more studies are necessary to draw consistent conclusions on safety and immunogenicity regarding AID patients, the HPV vaccination in our cohort of JDM patients was safe and immunogenic. No severe AEFV occurred, and disease activity remained stable or even improved during the study. Since the seropositivity against HPV16 and HPV18 was very high after the 3-dose schedule, this regimen should be recommended for JDM patients.

## Data Availability

Data sets generated and/or analyzed during the current study are available from the repository [ReBEC Brazilian Clinical Trial Registry], [http://www.ensaiosclinicos.gov.br/rg/RBR-9ypbtf/] and also from the corresponding author upon reasonable request.

## Abbreviations

AEFV: Adverse events following vaccination
AID: Autoimmune diseases
CMAS: Childhood myositis activity score
HC: Healthy controls
HPV: Human papillomavirus
JDM: Juvenile dermatomyositis
MMT: Manual muscle testing
NIP: Brazilian National Immunization Program
qHPV: Quadrivalent HPV vaccine
